# High serum HTATIP2/TIP30 level in serous ovarian cancer as prognostic or diagnostic marker

**DOI:** 10.1186/2047-783X-18-18

**Published:** 2013-06-24

**Authors:** Yakup Kumtepe, Zekai Halici, Ozlem Sengul, Celalettin Semih Kunak, Yasin Bayir, Nergiz Kilic, Elif Cadirci, Alparslan Pulur, Zafer Bayraktutan

**Affiliations:** 1 Department of Obstetrics and Gynecology, Ataturk University, Faculty of Medicine, Erzurum 25240, Turkey; 2 Department of Pharmacology, Faculty of Medicine, Ataturk University, Erzurum 25240, Turkey; 3 Department of Gynecology and Obstetrics, Yenimahalle Government Hospital, Ankara 06170 Yenimahalle, Turkey; 4 Department of Pharmacology, Faculty of Medicine, Giresun University, Giresun 28100, Turkey; 5 Department of Biochemistry, Faculty of Pharmacy, Ataturk University, Erzurum 25240, Turkey; 6 Department of Obstetrics and Gynecology, Faculty of Medicine, Kafkas University, Kars 36000, Turkey; 7 Department of Pharmacology, Faculty of Pharmacy, Ataturk University, Erzurum 25240, Turkey; 8 Department of Biochemistry, Erzurum Region Education and Research Hospital, Erzurum 25240, Turkey

**Keywords:** HTATIP2/TIP30, ovarian cancer, adenocarcinoma

## Abstract

**Background:**

Human HIV-1 TAT interactive protein 2 (HTATIP2/TIP30) is an evolutionarily conserved gene that is expressed ubiquitously in human tissues and some tumor tissues. This protein has been found to be associated with some gynecological cancers; as such, this study aimed to investigate blood HTATIP2/TIP30 levels in patients with ovarian cancer.

**Methods:**

Twenty-three women with ovarian cancer and 18 patients with various non-cancerous gynecological complaints (for example, dysfunctional uterine bleeding, fibroids, and urinary incontinence) were included in the study. The pathological diagnosis of ovarian cancer was adenocarcinoma. HTATIP2/TIP30 concentration in the patients’ blood samples was determined using ELISA kits.

**Results:**

The HTATIP2/TIP30 level was significantly higher in the cancer group than in the control group (1.84 ± 0.82 versus 0.57 ± 0.13 ng/ml, mean ± SD).

**Conclusions:**

We demonstrated the potential role of HTATIP2/TIP30 in ovarian cancer for the first time, thereby enlightening future studies targeting HTATIP2/TIP30 in ovarian cancer treatment, diagnosis, and prevention.

## Background

Ovarian cancer is the seventh most common cancer in women in terms of both incidence and mortality. The histologic subtypes of ovarian cancer are epithelioid (serous, endometrioid, mucinous, clear cell, and undifferentiated) and non-epithelioid [1]; the epithelioid subtype accounts for 90% of malignancies [2]. The onset is often insidious; the symptoms are vague and may mimic other conditions. This may lead to a delay in diagnosis, and currently, three-quarters of women with ovarian cancer are diagnosed when the disease has spread throughout the abdomen [3,4].

Many factors affect cell growth and related abnormal tumor growth in ovarian cancer. Epidermal growth factor receptor (EGFR) is also central to the promotion of cell growth and has a role in the development of cancer. Therefore, preventing EGFR activity is an attractive target for novel therapeutic agents. EGFR is a member of a family of similar molecules called the epidermal growth factor receptor family. This family also includes human epidermal growth factor receptor 2 (HER2/neu), Erb3, and Erb4. HER2/neu overexpression is related to poor outcome in breast and ovarian cancers [5-8]. A monoclonal antibody, trastuzumab (Herceptin), has been developed that binds to HER2/neu [5,6]. A 7.3% response rate was demonstrated, with no significant toxicity, in women with recurrent ovarian cancer treated with Herceptin [9]. In addition to these studies demonstrating the role of HER2/neu in gynecological cancers, Zhang *et al*. reported that overexpression of human HIV-1 TAT interactive protein 2 (HTATIP2/TIP30) is associated with HER2/neu status in breast cancer [10].

HTATIP2/TIP30 is an evolutionarily conserved gene that is expressed ubiquitously in human tissues and some tumor tissues. HTATIP2/TIP30 has been shown to be bound to the transcriptional activation domain of the human immunodeficiency virus (HIV-1) TAT and to act as a cofactor to specifically enhanced TAT-activated transcription [11]. The discovery of the roles of HTATIP2/TIP30 in apoptosis and tumor suppression, especially in controlling the expression of genes involved in apoptosis and metastasis suppression, indicates that there is a signal pathway facilitated by HTATIP2/TIP30 and its associated factors [12-17]. It also interacts with an estrogen receptor α (ERα)-interacting coactivator CIA and regulates ERα-mediated c-Myc transcription [18,19]. All of this information led us to hypothesize a potential role of HTATIP2/TIP30 in ovarian cancer.

The aim of our study was to investigate the levels of HTATIP2/TIP30 in serous ovarian cancers, using the enzyme-linked immunosorbent assay (ELISA) method, and to determine whether altered HTATIP2/TIP30 levels correlate with cancer occurrence.

## Methods

### Patients

Between March 2009 and February 2010, 34 patients who were suspected of having ovarian cancer and who visited Ataturk University, Faculty of Medicine, Department of Obstetrics and Gynecology, were evaluated in this study. Before surgery, whole abdominopelvic magnetic resonance imaging, vaginal ultrasound, and Ca-125 level testing were performed to determine the presence of tumors and ascites, according to our routine clinical approach, and 5 ml of blood were taken. This study was approved by the Local Human Ethics Committee of the Ministry of Health.

All of the patients underwent radical cytoreductive surgery to remove all visible tumors, including hysterectomy, bilateral salpingo-oophorectomy, appendectomy, infracolic omentectomy, and systemic pelvic and para-aortic lymphadenectomy. Based on the final pathology results, those confirmed to have serous ovarian cancer (n = 23, stages III and IV) were included in the final data analysis of the study group. After surgical intervention, the treatment of 11 patients with other cancer types (mucinous, n = 5; endometrioid, n = 4; clear cell, n = 1; and Brenner cell, n = 1) was continued with platinum-based chemotherapy, but they were excluded from the data analysis.

The 23 patients diagnosed with epithelial serous ovarian cancer (grade I, n = 4; grade II, n = 13; and grade III, n = 6) were chosen for the study group. During the same patient selection period, 18 patients with various non-cancerous gynecological complaints (dysfunctional uterine bleeding, n = 6; fibroids, n = 7; urinary incontinence, n = 3; and endometrioma, n = 2) who were referred to our polyclinics were chosen for the control group. The ages of the subjects in the control group were matched with the ages of the subjects in the study group. All procedures were reviewed and approved by the Institutional Ethics Committee on Human and Animal Research. All subjects were given an explanation regarding the aim of the study.

### Blood sampling

Blood samples were collected from these patients in biochemical tubes and immediately taken to the biochemistry laboratory, where they were centrifuged at 4,000 rpm for 10 minutes at 5°C, and sera was obtained. The sera were kept at −80°C until the biochemical measurements for HTATIP2/TIP30 evaluation were conducted.

### Biochemical analyses

HTATIP2/TIP30 concentration was determined using a commercially available ELISA kit (Cusabio Biotech Co., Ltd.(Incubator Building 4th floor, Wuhan University Science Park,No.te-1, Daxueyuan Road, Donghu Hi-Tech Development Area, Wuhan, Hubei Province 430223, P.R.China) catalog number: CSB-E14917H ELISA kit). For this kit, the minimum detectable dose of human HTATIP2 is typically less than 0.16 ng/ml. The sensitivity of this assay, or lower limit of detection (LLD), was defined as the lowest protein concentration that could be differentiated from zero. Intra-assay precision (precision within an assay) is CV% < 8%, and inter-assay precision (precision between assays) is CV% < 10%.

### Statistical analyses

The IBM SPSS Statistics 20 (IBM Corporation Software Group Route 100 Somers, NY 10589)computer program package was used for statistical calculations. The results were expressed as mean ± standard deviation (SD). Unpaired Student’s *t-*tests were used to analyze the significant differences in HTATIP2/TIP30 levels between the control and ovarian cancer groups.

## Results

The women’s age ranges were 42 to 81 years (mean age, 65 ± 24 years) in the study group and 39 to 78 years (mean age, 59 ± 22 years) in the control group. The mean parity was 4.2 (1 to 8) in the study group and 4.3 (2 to 8) in the control group. Only a few patients (n = 4) in the two groups combined were smokers.

**Figure 1:**
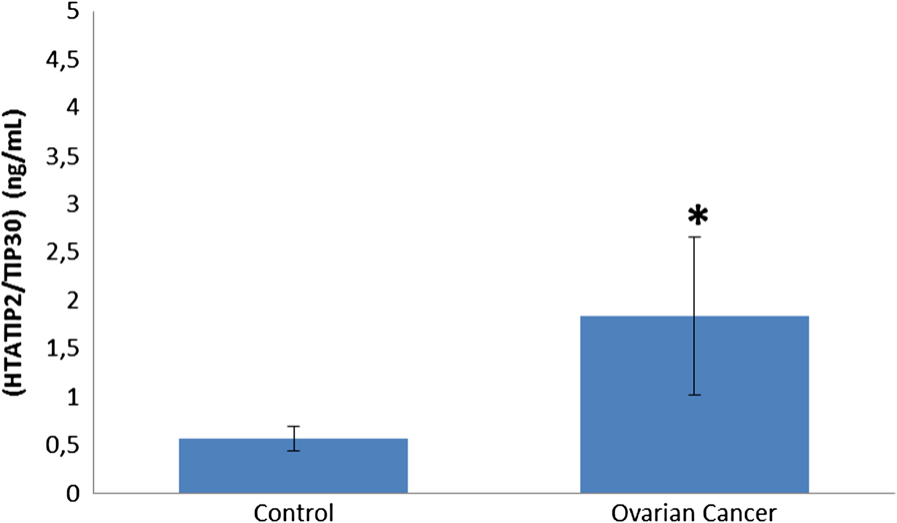
**Human HIV-1 TAT interactive protein 2 (HTATIP2/TIP30) in blood of patients with ovarian cancer and healthy controls.** *P < 0.05, unpaired *t*-test.

As seen in Figure 1, the HTATIP2/TIP30 level was significantly higher in the cancer group than in the control group (1.8363 ± 0.8177 (minimum: 0.82 to maximum: 3.67) versus 0.5659 ± 0.127 (minimum: 0.36 to maximum: 0.79) ng/ml, respectively).

## Discussion

Clinical studies regarding the status of HTATIP2/TIP30 expression in cancer patients are limited. The aim of this study was to demonstrate the involvement of HTATIP2/TIP30 in advanced epithelial serous ovarian cancer. Tong *et al*. [20] showed that downregulation of HTATIP2/TIP30 promoted metastasis in lung cancer. Downregulation of HTATIP2/TIP30 has also been observed in other cancer types, including melanoma, colon cancer, breast cancer, neuroblastoma, and hepatocellular carcinoma [13,14,16]. Several cell culture studies have demonstrated the role of HTATIP2/TIP30 in a number of tumor cell lines, including neuroblastoma, breast cancer, glioblastoma, and melanoma. In the present study, we found that HTATIP2/TIP30 levels were elevated in the serum samples of patients with ovarian cancer.

HTATIP2/TIP30 is an evolutionarily conserved gene that is expressed ubiquitously in human tissues and some tumor tissues [11]. It is known that there has been very significant progress in the development of anti-cancer agents, including tyrosine kinase inhibitors, angiogenesis inhibitors, and agents that interact with the cell cycle and cell death (apoptosis). HTATIP2/TIP30 displays a serine-threonine kinase activity that can phosphorylate the carboxyl terminal domain of RNA polymerase II in a TAT-dependent manner [12], suggesting that HTATIP2/TIP30 is a potential target for human cancer treatment. In addition, increasing evidence suggests that receptor tyrosine kinase activation participates in the oncogenic progression from the non-neoplastic mesothelial lining of the ovaries or the fallopian tube epithelium to epithelial ovarian cancer [21]. In light of these data, we can suggest that the increase in HTATIP2/TIP30 levels in ovarian cancer may be related to its intrinsic protein kinase activity.

The discovery of the roles of HTATIP2/TIP30 in apoptosis and tumor suppression, especially in controlling the expression of genes involved in apoptosis and metastasis suppression, indicates that there is a signal pathway facilitated by HTATIP2/TIP30 and its associated factors [12-17]. The literature shows that it is believed that the mechanism of HTATIP2/TIP30’s anti-metastatic properties is that it inhibits angiogenic properties of tumor cells and predisposes tumor cells to apoptosis [14,22]. Consistent with the role of HTATIP2/TIP30 in the suppression of tumor growth and metastasis via transcription mechanisms, studies have revealed that the ectopic expression of HTATIP2/TIP30 in tumor cell lines upregulates the expression of pro-apoptotic factors [12] and angiogenic inhibitors, and downregulates the expression of angiogenic stimulators [14]. In our study, the increased HTATIP2/TIP30 levels in the blood samples might be a response to increased angiogenesis in tumors.

HTATIP2/TIP30 also interacts with an estrogen receptor α-interacting coactivator and regulates ERα-mediated c-Myc transcription [18,19], suggesting that it has a role in ovarian cancer. Most of the effects of estrogen are facilitated by estrogen receptor α, which controls a number of hormone-responsive genes [23,24] including the c-Myc gene, which is important for cell proliferation [25-28]. The finding that HTATIP2/TIP30 acts as a negative regulator in both unliganded and liganded ERα-mediated c-Myc expression suggests that HTATIP2/TIP30 might regulate tumorigenesis and tissue development in ERα-targeted organs [29,30]. In addition, in our study, it was found that elevated HTATIP2/TIP30 levels might affect ovaries as ERα-targeted organs.

On the other hand, overexpression of HER2/neu, a member of the EGFR family, is related to poor outcomes in breast cancer [5,6] and ovarian cancer [7,8]. Studies are underway with oral agents that inhibit the tyrosine kinase activity associated with two oncogenes - EGFR and HER2/neu. In addition to these studies demonstrating the role of HER2/neu in gynecological cancers, Zhang *et al*. reported that overproduction of HTATIP2/TIP30 is associated with HER2/neu status in breast cancer [10]. All of these studies support our findings that HTATIP2/TIP30 has a potential role in ovarian cancer, and that HER2/neu expression may be related to increased HTATIP2/TIP30 levels.

## Conclusion

In this study, we demonstrated for the first time a potential role of HTATIP2/TIP30 in ovarian cancer which will enlighten future studies targeting HTATIP2/TIP30 in ovarian cancer treatment, diagnosis, and prevention. At the clinical level, our study is unable to suggest whether HTATIP2/TIP30 expression is suppressed or enhanced at the onset of ovarian cancers, when it is confined to the ovaries. Future clinical and experimental studies are required to determine the conclusive role of HTATIP2/TIP30 in ovarian cancer.

## Abbreviations

EGFR: Epidermal growth factor receptor; ELISA: Enzyme-linked immunosorbent assay; ERα: Estrogen receptor α; HER2/neu: Human epidermal growth factor receptor 2; HIV-1: Human immunodeficiency virus; HTATIP2/TIP30: Human HIV-1 TAT interactive protein 2; LLD: Lower limit of detection.

## Competing interest

The authors declare that they have no competing interest.

## Authors’ contributions

YK: Designed and controlled whole sections of the study with ZH. ZH: Designed and controlled whole sections of the study with YK. OS: Analyzed and clinically evaluated patients with NK and AP. CSK: Performed statistical analyses with EC. YB: Performed all biochemical analyses with ZB. NK: Analyzed and clinically evaluated patients with OS and AP. EC: Performed statistical analyses with CSK and wrote down manuscript in English. AP: Analyzed and clinically evaluated patients with OS and NK. ZB: Performed all biochemical analyses with YB. All authors read and approved the final manuscript.
